# Dysregulated arginine metabolism is associated with pro-tumor neutrophil polarization in liver cancer

**DOI:** 10.3389/fimmu.2025.1673665

**Published:** 2025-10-23

**Authors:** Xingchao Liu, Yinghui Zhang, Yangke He, Liang Liang

**Affiliations:** ^1^ Organ Transplantation Center, Sichuan Provincial People’s Hospital, School of Medicine, University of Electronic Science and Technology of China, Chengdu, China; ^2^ Department of Gastroenterology and Hepatology, Sichuan Provincial People’s Hospital, School of Medicine, University of Electronic Science and Technology of China, Chengdu, China; ^3^ Department of Oncology, Sichuan Provincial People’s Hospital, School of Medicine, University of Electronic Science and Technology of China, Chengdu, China

**Keywords:** hepatocellular carcinoma, tumor microenvironment, neutrophil heterogeneity, arginine metabolism, single-cell transcriptomics, PADI4

## Abstract

**Background:**

Liver hepatocellular carcinoma (LIHC) is a leading cause of cancer-related mortality, with an immunosuppressive tumor microenvironment (TME) contributing to therapeutic resistance. Although neutrophils are recognized as key regulators of LIHC progression, their functional heterogeneity and metabolic drivers are not yet fully understood.

**Methods:**

We integrated bulk RNA sequencing (RNA-seq) data from The Cancer Genome Atlas (TCGA) and the Gene Expression Omnibus (GEO) database (GSE39791) alongside scRNA-seq data from GSE149614 and GSE290925. Neutrophils were annotated based on specific marker genes (FCGR3B, CSF3R) and classified into three metabolic states: high arginine state (HAS), intermediate arginine state (DTAS), and low arginine state (LAS) using arginine metabolism-related gene sets. Differentiation trajectories were reconstructed via CytoTRACE and monocle2. Intercellular communication was analyzed using CellChat, while machine learning, incorporating seven different algorithms, was applied to identify key regulatory genes.

**Results:**

scRNA-seq analysis revealed three distinct neutrophil subgroups: high (HAS), intermediate (DTAS), and low (LAS) arginine metabolism states. The proportion of LAS neutrophils was significantly enriched in tumor tissues compared to normal tissues (p < 0.001). Trajectory analysis indicated that LAS neutrophils exhibited a less differentiated state. From this landscape, ATP11B and PADI4 were identified as key genes, with PADI4 expression being approximately 3-fold higher in HAS compared to LAS neutrophils. Functional studies demonstrated that silencing PADI4 in LIHC cell lines inhibited cell proliferation by approximately 50% at 96 hours, increased apoptosis by 2-fold, and reduced cell invasion by 50%.

**Conclusions:**

Arginine metabolism shapes neutrophil polarization in the LIHC TME. Targeting metabolic pathways may provide new therapeutic strategies to modulate the immune landscape and improve patient outcomes.

## Introduction

Liver hepatocellular carcinoma (LIHC) is the sixth most common cancer globally and the third leading cause of cancer-related mortality (1). It typically arises in the setting of chronic liver diseases, with cirrhosis present in over 80% of cases (2). Key risk factors include chronic hepatitis B and C infections, alcoholic liver disease, metabolic syndrome, and exposure to aflatoxins (3, 4). These factors contribute to liver inflammation and fibrosis, both of which are critical precursors to tumorigenesis (5). Despite advancements in diagnostic imaging, such as multiphase CT, MRI, and serum tumor markers like alpha-fetoprotein (AFP), early detection of LIHC remains challenging, with many cases being diagnosed at advanced stages (6, 7). While early-stage LIHC can be treated effectively through surgical resection, transplantation, or ablation, survival rates for advanced stages remain poor, primarily due to the limited efficacy of current therapies, including immune checkpoint inhibitors and multi-kinase inhibitors (8–10). This is largely attributed to the immunosuppressive and fibrotic TME, which impedes effective immune responses and treatment outcomes (11, 12). Therefore, gaining a deeper understanding of the TME and the mechanisms underlying tumor progression is crucial for the development of more effective therapeutic strategies.

Chronic inflammation is a hallmark of all etiologies of chronic liver disease and plays a pivotal role in tumor initiation, progression, and metastasis (13). The immune microenvironment of the liver is uniquely tolerogenic due to the constant influx of inflammatory mediators from the portal circulation (14). This environment fosters the development of pre-neoplastic lesions that evade immune surveillance, ultimately leading to hepatocellular carcinoma (HCC) (15). Neutrophils, as the first responders to infection, inflammation, and tissue damage, are essential mediators of the innate immune response (16). They perform antimicrobial and inflammatory functions through mechanisms such as phagocytosis, degranulation, release of neutrophil extracellular traps (NETs), and antigen presentation (17, 18). Neutrophils are also key contributors to chronic inflammation and represent a significant component of the immune infiltrate in both chronic liver disease and HCC (19, 20). Tumor-associated neutrophils (TANs) exhibit remarkable functional plasticity, adapting to the metabolic and inflammatory cues present in the TME (21, 22). Critically, this functional plasticity is underpinned by profound metabolic reprogramming, which remains a relatively unexplored layer of regulation in LIHC.

The complexity of the immune system is not only reflected in its cellular diversity and intricate signaling networks but also in its close integration with metabolic processes (23). Immune responses require significant metabolic reprogramming to support cell proliferation, differentiation, and effector functions (24). Therefore, the metabolic microenvironment of the liver profoundly influences immune cell activity and functionality (25). Arginine (Arg), a semi-essential amino acid, plays a particularly important role during immune responses (26). In immune cells, Arg is metabolized by arginase-1 (Arg1) or arginase-2 (Arg2) to produce urea and L-ornithine (Orn), or it is utilized for protein biosynthesis (27). Nitric oxide synthases use Arg to generate nitric oxide, a key antimicrobial and signaling molecule (28). Arginase-1 activity has long been recognized as an important immunoregulatory mechanism, particularly in M2 macrophages and MDSCs within the tumor context (29). In neutrophils, arginine metabolism is altered, with a marked upregulation of ARG1 (30). This upregulation is driven by the TME and is particularly prominent in the formation of MDSCs, which are key mediators of immune suppression in tumors (31). Moreover, ARG1 activity is linked to the formation of NETs, which further promote immune evasion and tumor progression (32).

Neutrophils can be classified into two main phenotypes: the antitumor N1 phenotype and the protumor N2 phenotype. N1 neutrophils directly kill tumor cells via the production of reactive oxygen species (ROS) and reactive nitrogen species (RNS), whereas N2 neutrophils promote tumor progression by facilitating angiogenesis, metastasis, and immune suppression (33). Additionally, polymorphonuclear myeloid-derived suppressor cells (PMN-MDSCs), a subset of immature neutrophils, contribute to tumor progression through immune suppression, tissue remodeling, and angiogenesis (34, 35). Recent advances in scRNA-seq have unveiled the considerable heterogeneity of TANs, revealing distinct transcriptomic signatures associated with disease progression and patient prognosis (36). Understanding the molecular mechanisms governing neutrophil polarization and reprogramming in the TME is crucial for identifying new therapeutic strategies that can modulate the immune landscape of LIHC.

This study aims to explore the role of arginine metabolism in neutrophil polarization within the LIHC TME. While previous single-cell studies have described the heterogeneity of TANs, the metabolic drivers underlying this diversity remain poorly understood. Here, we leverage scRNA-seq not merely to catalog cell states, but to dissect how arginine metabolism reprograms neutrophil differentiation, functional plasticity, and their contribution to tumor progression. Ultimately, our research seeks to identify novel biomarkers and therapeutic targets that can modulate the immune landscape of LIHC, with a focus on improving patient outcomes through targeted metabolic and immune interventions.

## Materials and methods

### Data acquisition and processing

Transcriptomic data for LIHC were obtained from the Xena database (https://xena.ucsc.edu/), comprising RNA expression profiles and corresponding clinical data for 374 tumor samples and 50 adjacent normal samples. The data were normalized to Transcripts Per Million (TPM) and log_2_-transformed for subsequent analysis. Additional validation was performed using RNA data from the GEO database (https://www.ncbi.nlm.nih.gov/gds/?term=) (GSE39791), which includes 72 tumor and 72 adjacent normal samples.

Single-cell RNA sequencing datasets were retrieved from GEO database (GSE149614 and GSE290925), including 8 adjacent normal and 12 tumor samples. Data processing and analysis were performed using R, and Seurat was employed for quality control, normalization, and clustering. Cells were filtered based on the following criteria: mitochondrial gene content <20%, blood cell content <3%. To exclude potential doublets and multiplets, we enforced an upper threshold for both UMI counts (20,000) and the number of genes detected (6,000). A lower threshold (UMI > 200, genes > 200) was applied to remove empty droplets and low-quality cells. Normalization was performed using NormalizeData, and high-variable genes were identified using FindVariableFeatures (top 2,000 genes). Batch effect correction was implemented using Harmony. For dimensionality reduction and clustering, UMAP and the Louvain algorithm were applied, respectively. Differential gene expression between clusters was identified using FindAllMarkers with criteria of p-value <0.05, log_2_ fold change >0.25, and expression ratio >0.1.

Arginine metabolism-related gene sets were obtained from the Molecular Signatures Database (MsigDB), specifically the GOBP_ARGININE_METABOLIC_PROCESS.v2025.1.Hs.gmt file (https://www.gsea-msigdb.org/gsea/msigdb/human/geneset/GOBP_ARGININE_METABOLIC_PROCESS.html). This gene set was used to calculate arginine metabolism scores for the single-cell data.

All the data in this study were sourced from a public database and no additional ethical approval was required. This study adhered to relevant regulations in the acquisition and processing of data.

### Cell annotation

Cells were annotated based on canonical marker genes for specific cell types. Hepatocytes were identified by markers EPCAM, KRT18, KRT19, and ALB; fibroblasts by DCN, THY1, COL1A1, and COL1A2; endothelial cells by PECAM1, CLDN5, FLT1, and RAMP2; T cells by CD3D, CD3E, CD3G, and TRAC; NK cells by NKG7, GNLY, NCAM1, and KLRD1; B cells by CD79A, IGHM, IGHG3, and IGHA2; plasma cells by JCHAIN; myeloid cells by LYZ, MARCO, CD68, and FCGR3A; mast cells by KIT, MS4A2, GATA2; and neutrophils by FCGR3B and CSF3R. UMAP and bubble plots were generated to visualize the expression of these markers across the dataset.

### Neutrophil subgroup analysis

Neutrophils were isolated from the dataset based on the expression of neutrophil-specific marker genes (“FCGR3B”, “CSF3R”) using the Seurat subset function. The arginine metabolism scores for each neutrophil were calculated using the UCell algorithm, based on the GOBP_ARGININE_METABOLIC_PROCESS gene set. The neutrophil population was then divided into three distinct subgroups based on their arginine metabolism scores: HAS, DTAS, and LAS. These cutoff values were determined empirically by identifying significant points of change in the distribution of arginine metabolism scores.

### CytoTRACE analysis

To assess the differentiation potential of neutrophil subgroups, the CytoTRACE method was applied. CytoTRACE is a computational tool that estimates the differentiation potential of single cells based on gene expression data. For each neutrophil subgroup, the CytoTRACE score was calculated, which reflects the relative differentiation potential of individual cells.

### MiloR and Ro/e analysis

To quantitatively assess the spatial distribution and differential abundance of neutrophil subgroups in tumor and adjacent normal tissues, we performed MiloR and Ro/e analyses on the single-cell data.

MiloR analysis was employed to identify statistically significant differences in the local cellular neighborhoods of neutrophil subgroups between conditions (tumor vs. normal). Briefly, we first constructed a k-nearest neighbor (KNN) graph of all cells in the integrated dataset. The value of k was set to 50 to define a sufficiently large local neighborhood. Neighborhoods were then sampled by randomly selecting 100 representative index cells. For each neutrophil subgroup (HAS, DTAS, LAS), we tested for differential abundance between tumor and normal tissues within these neighborhoods using a negative binomial generalized linear model (GLM). A false discovery rate (FDR) of 5% was applied to correct for multiple hypothesis testing.

Ro/e (Ratio of observed to expected) analysis was used to quantify the enrichment or depletion of cell type interactions beyond random chance. We first constructed a contingency table of cell type counts across the KNN graph (with k=50). The “observed” count was the actual number of edges between a neutrophil subgroup and every other cell type. The “expected” count was calculated based on the product of their overall abundances, representing the number of edges expected if cell types were randomly distributed. The Ro/e value was then calculated as Ro/e = Observed/Expected. An Ro/e value > 1.1 was interpreted as a significant attraction (enrichment) between two cell types, while a value < 0.9 was interpreted as a significant repulsion (depletion). These analyses were performed separately for the tumor and normal tissue microenvironments to reveal context-specific interaction patterns.

### Pagwas analysis

To further investigate the functional role of neutrophil subgroups in the context of arginine metabolism, Pagwas analysis was performed. Pagwas is a pathway-based analysis tool that integrates gene expression profiles with pathway-specific scores, allowing for the exploration of biological pathways associated with arginine metabolism in different neutrophil subgroups. The analysis evaluated the relationship between arginine metabolism scores and TRS scores, offering insights into the activation of key biological pathways in neutrophils with varying arginine metabolic states.

### Pseudotime analysis

Pseudotime analysis of neutrophil subgroups was performed using the monocle2 package. The DDRTree algorithm was used for dimensionality reduction to infer the differentiation trajectory of neutrophils. Default parameters were used for all other steps, and the resulting pseudotime trajectories were visualized to assess the differentiation states of neutrophils from HAS, DTAS, and LAS.

### Cell-cell communication analysis

Cell-cell communication between neutrophils and other cell types was analyzed using the CellChat package. Normalized gene expression matrices were imported into CellChat to construct the communication networks. Overexpressed genes and interactions were identified using identifyOverExpressedGenes and identifyOverExpressedInteraction functions. Potential ligand-receptor interactions were predicted using computeCommunProb and filterCommunication. Communication networks were visualized using the aggregateNet function.

### Gene set scoring

Arginine metabolism scores for individual neutrophils were calculated using four different methods: AUCell, UCell, AddModuleScore, and Singscore. These methods were employed to compute a comprehensive metabolism score for each cell, which was used to categorize neutrophils into the three subgroups (HAS, DTAS, and LAS). These scores were correlated with functional pathways to assess potential biological impacts.

### Machine learning-based gene identification

To identify key genes associated with neutrophil polarization influenced by arginine metabolism, seven machine learning algorithms were applied: Decision Trees, Random Forests, GBM, Boruta, ABESS, XGBoost, and LASSO. Results from all models were integrated using Upset analysis, identifying genes consistently selected across algorithms.

### Clinical sample collection and processing

Primary tumor tissues and matched adjacent normal tissues (≥3 cm from the tumor margin) were collected from five liver hepatocellular carcinoma (LIHC) patients who underwent surgical resection at Sichuan Provincial People’s Hospital between May 2022 and April 2024. Fresh samples were snap-frozen in liquid nitrogen and stored at −80 °C for subsequent analysis. The study was approved by the Institutional Ethics Committee of Sichuan Provincial People’s Hospital, and written informed consent was obtained from all participants.

### RNA extraction and qRT-PCR analysis

Total RNA was extracted from tissue samples using TRIzol reagent (Invitrogen, USA) following the manufacturer’s protocol. RNA concentration and purity were assessed with a NanoDrop 2000 spectrophotometer (Thermo Fisher Scientific, USA). Complementary DNA (cDNA) was synthesized using the PrimeScript RT Reagent Kit (Takara, Japan). qRT-PCR was performed on a QuantStudio 5 Real-Time PCR System (Applied Biosystems, USA) with SYBR Premix Ex Taq (Takara, Japan). PADI4 mRNA expression was normalized to GAPDH using the 2^−ΔΔCt method, and reactions were conducted in triplicate.

### Cell culture and characterization

Human liver cancer cell lines (HuH-7, Hep G2, SNU-886, Hep 3B2.1-7, SNU-387) and the non-tumorigenic human liver cell line LO2 were obtained from authenticated cell banks and verified by STR profiling. All cell lines were mycoplasma-free. Cells were cultured in Dulbecco’s Modified Eagle Medium (DMEM; Gibco, USA) with 10% fetal bovine serum (FBS; Gibco) and 1% penicillin-streptomycin, maintained at 37 °C in a 5% CO_2_ incubator. When cells reached 80% confluency, total RNA was extracted and PADI4 mRNA levels were assessed by qRT-PCR in triplicate using independent biological replicates.

### siRNA transfection

siRNA targeting PADI4 and a non-targeting control siRNA were synthesized and dissolved in nuclease-free water at a final concentration of 10 μM. SNU-886 and SNU-387 cells were seeded in 6-well plates (2 × 10^5^ cells/well) and transfected with 50 nM siRNA and 5 μL of Lipofectamine 3000 reagent (Invitrogen, USA) in Opti-MEM medium (Gibco, USA). After 6 hours, the transfection medium was replaced with complete growth medium. RNA was harvested 48 hours post-transfection, and knockdown efficiency was confirmed by qRT-PCR, showing a >70% reduction in PADI4 expression (p < 0.01, Student’s t-test). All experiments were independently repeated three times.

### Cell proliferation assay (CCK-8)

Post-transfection, cells were seeded in 96-well plates (3 × 10³ cells/well) in quintuplicate. Cell proliferation was assessed at 24, 48, 72, and 96 hours post-transfection using the CCK-8 assay (Dojindo, Japan). At each time point, 10 μL of CCK-8 reagent was added to each well and incubated for 2 hours at 37 °C. Absorbance at 450 nm was measured using a Synergy H1 microplate reader (BioTek, USA). Relative cell viability was calculated by normalizing absorbance to the 0-hour baseline, and proliferation curves were plotted.

### Apoptosis assay (flow cytometry)

Apoptosis was analyzed 48 hours post-transfection using the Annexin V-FITC/PI Apoptosis Detection Kit (BD Biosciences, USA). Cells were collected, washed with cold PBS, and stained according to the manufacturer’s protocol. Flow cytometry was performed using a BD FACSVerse flow cytometer (BD Biosciences), and data were analyzed with FlowJo software (version 10). The proportions of early apoptotic (Annexin V^+^/PI^-^) and late apoptotic (Annexin V^+^/PI^+^) cells were quantified.

### Migration and invasion assays

Cell migration and invasion were assessed using 24-well Transwell chambers with 8 μm pore-size membranes (Corning, USA). For migration assays, 5 × 10^4^ cells in 200 μL serum-free DMEM were added to the upper chamber, and 600 μL DMEM supplemented with 10% FBS was placed in the lower chamber. After 24 hours, non-migrated cells were removed with a cotton swab, and migrated cells were fixed with 4% paraformaldehyde, stained with 0.1% crystal violet, and counted in five randomly selected fields under a microscope.

For invasion assays, Transwell membranes were pre-coated with Matrigel (Corning, USA) diluted 1:8 in DMEM, incubated for 4 hours at 37 °C, and then subjected to the same procedure as the migration assay.

### Western blotting

Total protein was extracted using RIPA buffer (Beyotime, China) supplemented with protease inhibitors (Roche, Switzerland). Protein concentrations were measured using a BCA protein assay kit (Thermo Fisher Scientific, USA). Equal amounts of protein (30 μg) were separated by 10% SDS-PAGE, transferred to PVDF membranes (Millipore, USA), and blocked with 5% non-fat milk in TBST for 1 hour. Membranes were incubated overnight at 4 °C with primary antibodies: anti-cleaved Caspase-3 (1:1000, #9664), anti-E-cadherin (1:2000, #3195), anti-Bcl-2 (1:1000, #15071), anti-Vimentin (1:1000, #5741), and anti-β-actin (1:5000, #4970) (all from Cell Signaling Technology, USA). After washing, membranes were incubated with HRP-conjugated secondary antibodies (1:5000) for 1 hour. Protein bands were visualized using enhanced chemiluminescence (ECL, Millipore) and quantified using ImageJ software.

### Statistical analysis

All statistical analyses were conducted using R (version 4.1.3). The Pearson correlation coefficient was calculated to assess relationships between continuous variables. The Chi-squared test was applied to categorical variables, and the Wilcoxon rank-sum test was used for comparisons of continuous variables. Survival analyses were performed using the survival and survminer packages, with optimal cutoff values determined using survminer. Kaplan-Meier curves and Cox regression analysis were used to assess the prognostic significance of arginine metabolism states and identified genes. Statistical significance was set at a *P* value of less than 0.05. In figures, asterisks denote statistical significance as follows: *P < 0.05; **P < 0.01; ***P < 0.001; ****P < 0.0001; "ns" indicates not significant (P ≥ 0.05).

## Results

### Characterization of the tumor microenvironment in LIHC using scRNA-seq

**Figure 1:**
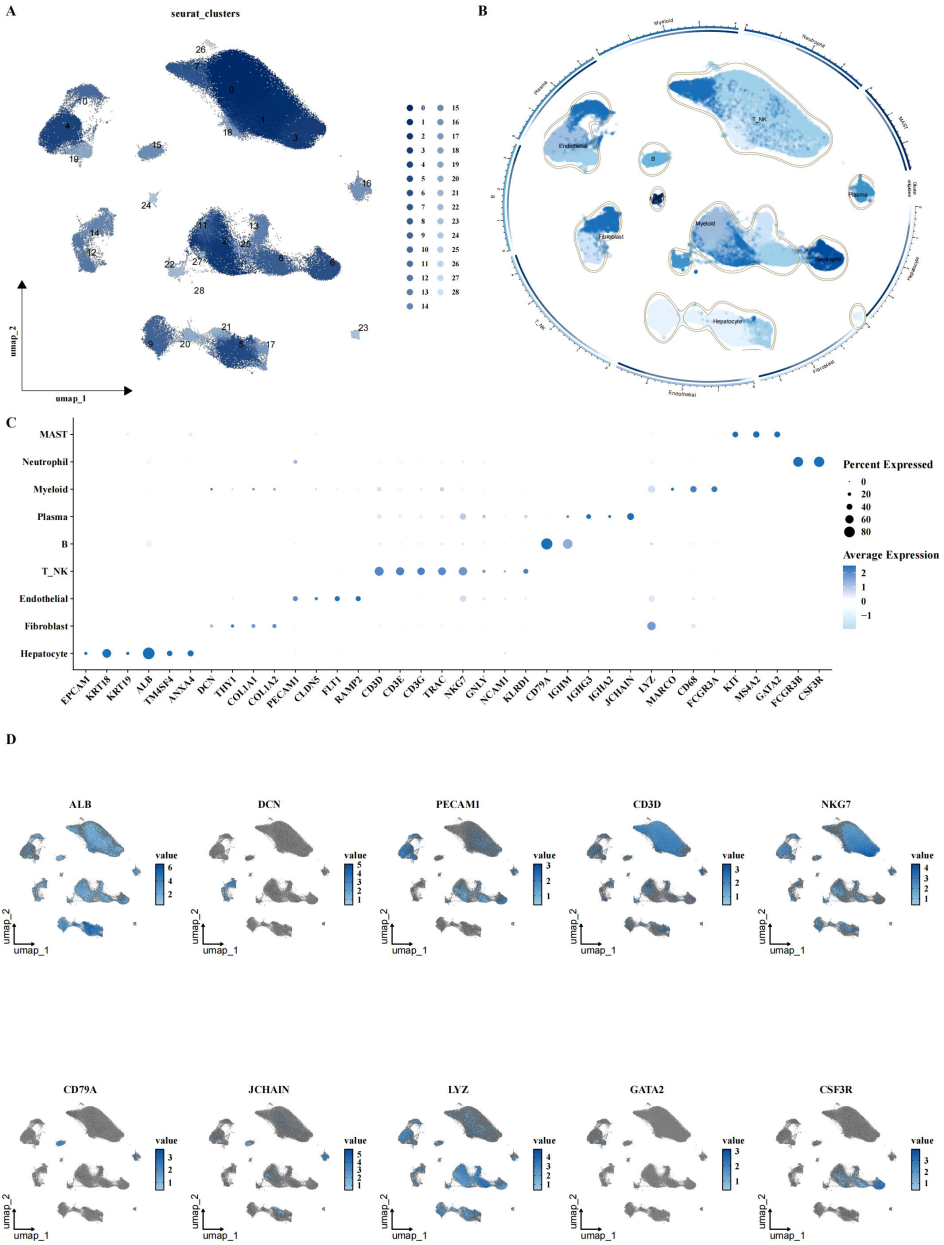
Single-cell characterization of LIHC cellular landscape. **(A, B)** UMAP plots showing cell clustering and annotation results, identifying 31 clusters with 9 major cell types, including hepatocytes, fibroblasts, endothelial cells, T/NK cells, B cells, plasma cells, myeloid cells, mast cells, and neutrophils. **(C, D)** Expression of canonical cell markers across different cell types, visualized by bubble plot and UMAP, indicating the expression levels of specific markers for each cell type.

To investigate the cellular landscape of LIHC, we performed scRNA-seq analysis on 8 adjacent normal and 12 tumor samples. After quality control and normalization, a total of 160,566 cells across 29 clusters were identified. Cell type annotation was performed based on canonical marker genes, resulting in the classification of cells into nine major types: hepatocytes, fibroblasts, endothelial cells, T cells, NK cells, B cells, plasma cells, myeloid cells, and neutrophils (**Figures 1A, B**). The expression of specific cell type markers was visualized using UMAP (**Figures 1C, D**), confirming accurate cell type identification.

### Altered arginine metabolism in the tumor microenvironment of LIHC

**Figure 2:**
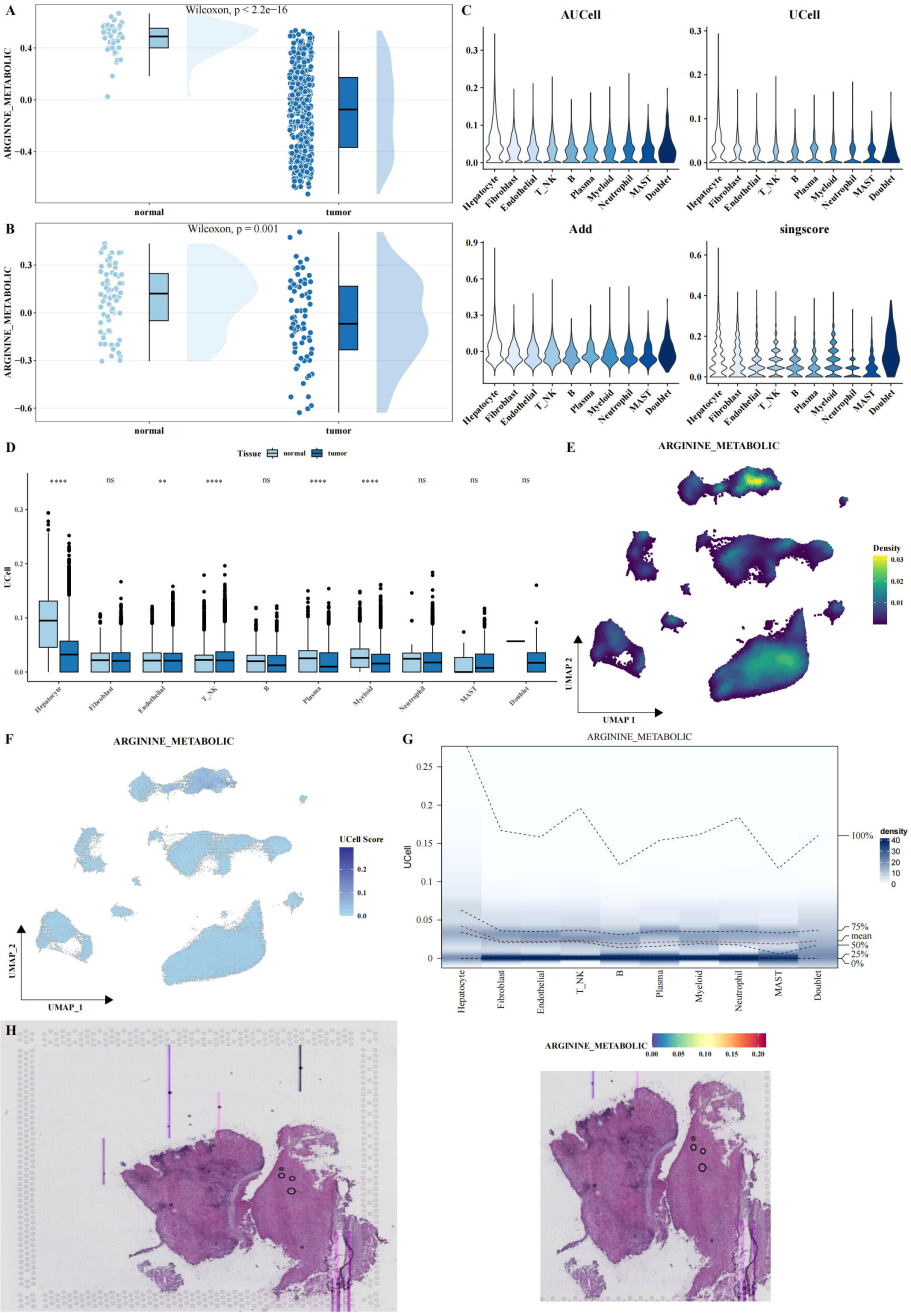
Functional analysis of arginine metabolism in LIHC. **(A, B)** Rain cloud plots showing significant differences in arginine metabolism scores between tumor and adjacent normal tissues in two bulk datasets (TCGA and GSE39791). **(C)** Violin plots showing arginine metabolism scores calculated using four different algorithms (AUCell, UCell, AddModuleScore, and Singscore) in single-cell data. **(D)** Box plots comparing arginine metabolism scores between tumor and adjacent normal tissues for each cell type, calculated using UCell. **(E-G)** UMAP density, UMAP, and probability density heatmaps illustrating the distribution of arginine metabolism scores across single-cell data. **(H)** HE and arginine metabolism score heatmaps in spatial transcriptomics data, validating the expression patterns of arginine metabolism in tissue samples. Asterisks denote statistical significance as follows: **P < 0.01; ****P < 0.0001; "ns" indicates not significant (P ≥ 0.05).

To explore the role of arginine metabolism in LIHC, we first calculated the arginine metabolism scores for both bulk RNA-seq and scRNA-seq datasets using the ssGSEA algorithm. In the bulk datasets, we observed a significant decrease in arginine metabolism scores in tumor tissues compared to adjacent normal tissues (**Figures 2A, B**). Next, we calculated the arginine metabolism scores for individual cells within the scRNA-seq dataset using four different methods: AUCell, UCell, AddModuleScore, and Singscore. While no significant differences were observed between the scores of different cell subtypes (**Figure 2C**), there were notable differences in the scores of specific cell subpopulations between tumor and adjacent normal tissues. In tumor tissues, hepatocytes, fibroblasts, endothelial cells, NK cells, plasma cells, and myeloid cells exhibited significantly lower arginine metabolism scores compared to adjacent normal tissues (**Figure 2D**). Among all cell types, hepatocytes displayed the highest arginine metabolism scores, both in tumor and normal tissues (**Figures 2E–G**). These findings were consistent across all cell types, highlighting the prominent role of hepatocytes in arginine metabolism in the liver cancer microenvironment. Similar results were observed in spatial transcriptomics data, further validating the robustness of these findings (**Figure 2H**).

### Impact of arginine metabolism on neutrophil subgroups and their differentiation in LIHC

**Figure 3:**
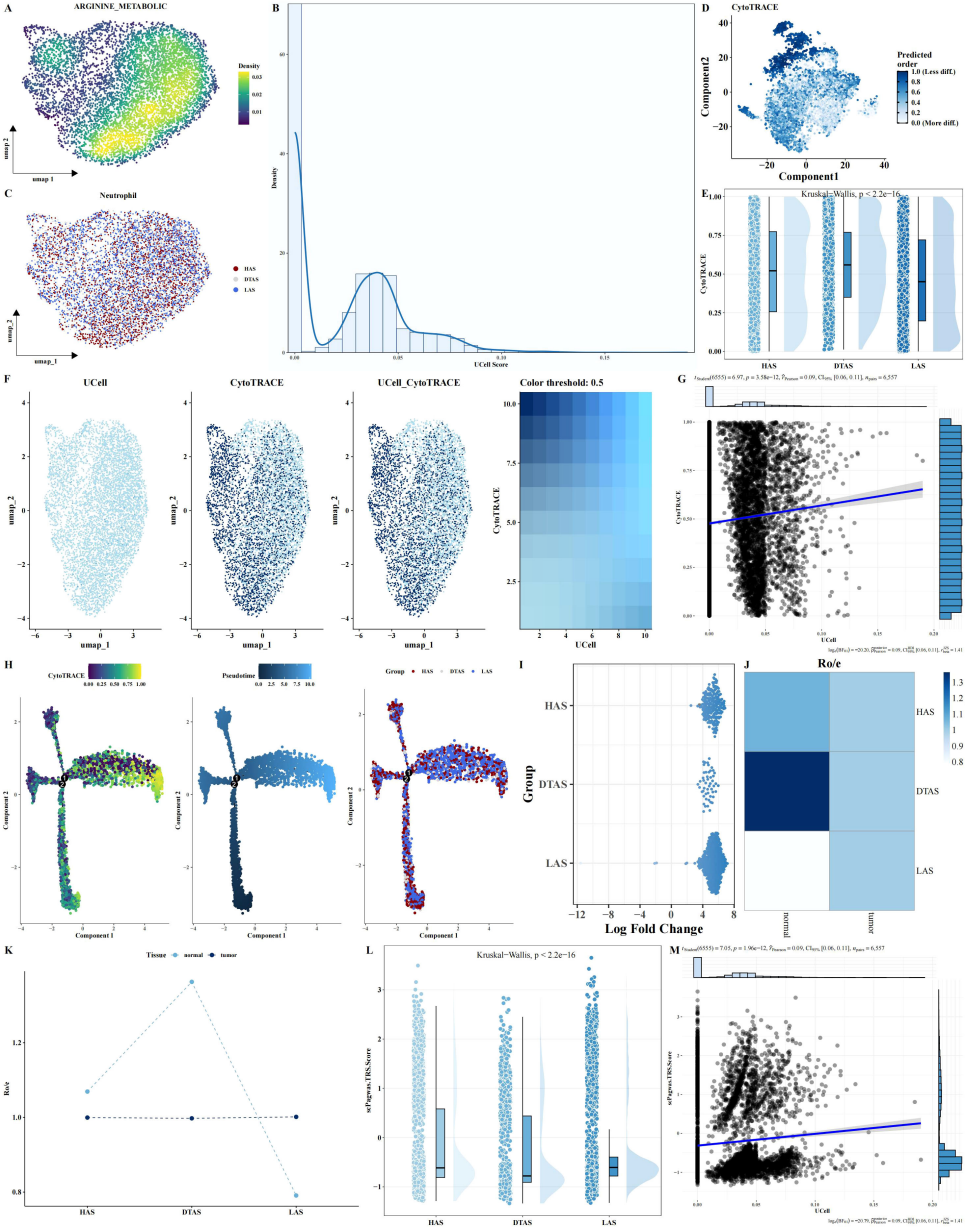
Neutrophil subgroup analysis based on arginine metabolism. **(A–C)** UMAP plots of neutrophils, showing arginine metabolism scores and cell grouping into HAS, DTAS, and LAS. **(D, E)** CytoTRACE-predicted differentiation potential scatter plots and rain cloud plots comparing differentiation across the three neutrophil groups. **(F, G)** FeaturePlot of differentiation potential and arginine metabolism score, showing a positive correlation between the two parameters. **(H)** Monocle2 trajectory analysis UMAP plot of neutrophils, visualizing differentiation trajectories based on arginine metabolism scores. **(I–K)** MiloR and Ro/e analyses, showing spatial distribution and enrichment of neutrophil subgroups in tumor versus adjacent normal tissues. **(L, M)** scPagwas analysis showing the correlation between arginine metabolism scores and TRS scores.

Although no significant differences were observed in the arginine metabolism scores of neutrophils between tumor and adjacent normal tissues, neutrophils are known to be highly heterogeneous cells, and their polarization can be influenced by metabolic states. To further investigate this, neutrophils were isolated from the scRNA-seq dataset. Arginine metabolism scores for individual neutrophils were calculated using the UCell algorithm. Neutrophils were then classified into three distinct subgroups based on their arginine metabolism scores: HAS, DTAS, and LAS (**Figures 3A–C**). While no obvious differences were observed across tumor and normal tissues at the overall neutrophil level, the classification into subgroups revealed significant differences in functional properties. To assess the differentiation potential of each subgroup, we applied CytoTRACE, a software tool for predicting cell differentiation trajectories. The results indicated that neutrophils in the LAS subgroup had the lowest CytoTRACE scores, suggesting a more undifferentiated state compared to the HAS and DTAS subgroups (**Figures 3D, E**). This finding suggests that neutrophils in the LAS state are less differentiated and may represent a more “naïve” phenotype, whereas neutrophils in the HAS state may be more functionally mature. Further analysis of the distribution of arginine metabolism scores and differentiation potential within neutrophil subgroups showed a significant positive correlation between arginine metabolism scores and CytoTRACE scores (**Figures 3F, G**). We also performed monocle2 trajectory analysis to infer the differentiation pathways of neutrophils. The results revealed distinct trajectories for the neutrophil subgroups, with HAS neutrophils occupying an earlier point in the differentiation trajectory, while LAS neutrophils were positioned later (**Figure 3H**). To explore the distribution of these subgroups across different tissue types, we performed MiloR and Ro/e analyses. These analyses showed that HAS neutrophils were enriched in normal tissues, while LAS neutrophils were more prominent in tumor tissues (**Figures 3I–K**), highlighting the role of arginine metabolism in neutrophil polarization within the tumor microenvironment. Finally, Pagwas analysis showed a significant positive correlation between arginine metabolism scores and TRS scores (**Figures 3L, M**), suggesting that arginine metabolism influences neutrophil polarization and may contribute to the functional differences observed between tumor and normal neutrophils.

### Distinct cell-cell interactions and pathway enrichments in neutrophil subgroups of LIHC

**Figure 4:**
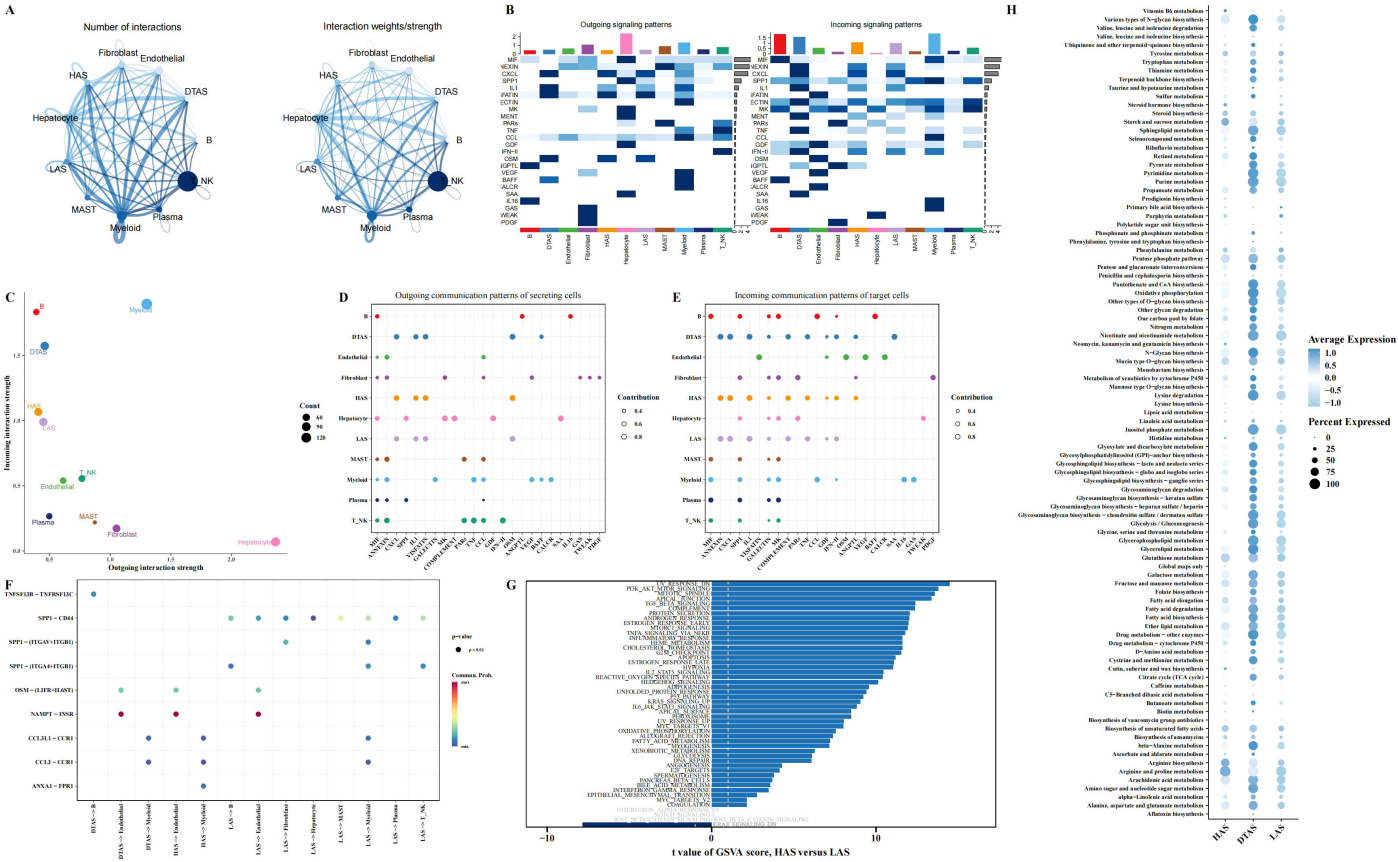
Intercellular communication and functional differences in neutrophil subgroups. **(A)** Communication networks between cell types, with the line thickness on the left representing the count of interactions and the line thickness on the right representing communication strength (weight). **(B)** Heatmaps illustrating the outgoing (left) and incoming (right) signaling patterns across different cell types. **(C)** Scatter plot comparing outgoing interaction strength (x-axis) versus incoming interaction strength (y-axis) for each cell type. **(D, E)** Bubble plots showing the incoming and outgoing communication of each cell type in various signaling pathways, highlighting key intercellular interactions. **(F)** Communication bubble plot comparing neutrophils (HAS, DTAS, LAS) with other cell types, showing differences in signaling interactions. **(G)** Bar plots illustrating functional differences in 50 hallmark pathways between HAS and LAS neutrophils. **(H)** Bubble plot showing the expression of metabolic-related pathways from the KEGG database in HAS, DTAS, and LAS neutrophils.

To explore the potential communication between neutrophils and other cell types within the tumor microenvironment, we used the CellChat package to construct a cell-cell interaction network. This analysis revealed notable differences in communication patterns between the three neutrophil subgroups (HAS, DTAS, and LAS) and other cell types (**Figures 4A, B**). Compared to HAS and LAS, DTAS neutrophils received significantly more incoming signals from surrounding cells (**Figure 4C**). In terms of signaling, all three neutrophil subgroups released common signals such as CXCL, IL1, VISFATIN, and OSM, while also receiving a shared set of signals including ANNEXIN, CXCL, and IL1 (**Figures 4D, E**). Notably, DTAS neutrophils also received SAA signals, which were absent in the HAS and LAS subgroups (**Figures 4D, E**). Additionally, strong communication was observed between neutrophils and endothelial cells, specifically through the NAMPT-INSR interaction, a key signaling axis in tumor vascularization (**Figure 4F**). Interestingly, LAS neutrophils exhibited a unique interaction with other cell types through SPP1-CD44 signaling, which was not seen in HAS or DTAS neutrophils (**Figure 4F**). We also examined pathway enrichment using 50 hallmark pathways to identify differential activity between the HAS and LAS subgroups. The results showed that HAS neutrophils exhibited higher activity in pathways such as PI3K-AKT-MTOR signaling, TGF-beta signaling, and several others associated with immune responses and cell survival (**Figure 4G**). In contrast, LAS neutrophils displayed elevated activity in the KRAS signaling pathway (**Figure 4G**). Furthermore, using KEGG metabolic pathways, we observed that DTAS neutrophils exhibited more active metabolic signaling, suggesting that they may be more metabolically active compared to the other two subgroups (**Figure 4H**).

### Gene module profiling and differential expression in neutrophil subgroups

**Figure 5:**
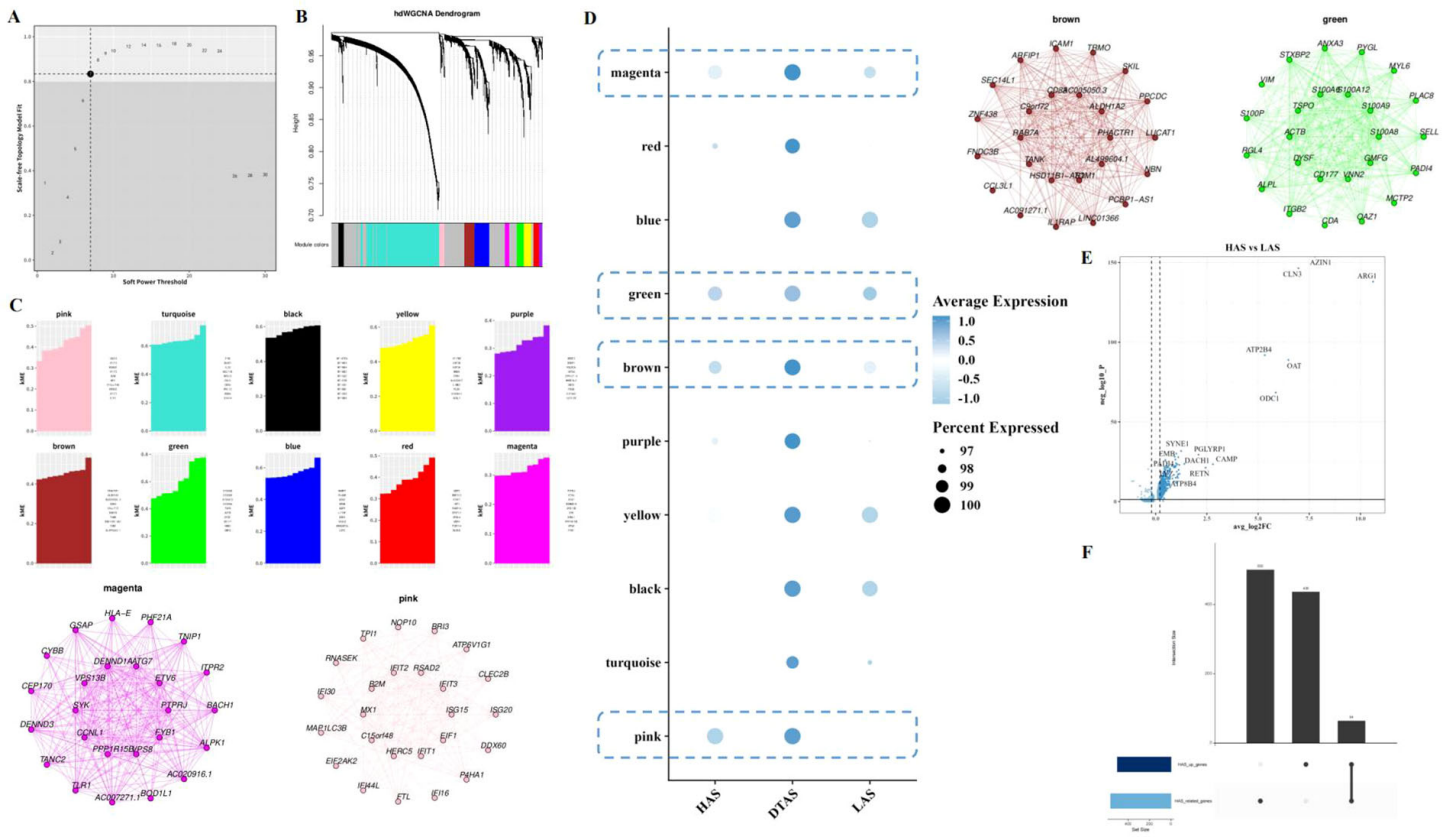
Gene module analysis in neutrophil subgroups. **(A)** Scatter plot selecting power as 7 for the hdWGCNA analysis, determining the optimal power parameter for module detection. **(B)** Hierarchical clustering dendrogram of 10 gene modules. **(C)** kME (module membership) plot for each of the identified gene modules. **(D)** Bubble plot showing the expression of 10 gene modules across the three neutrophil subgroups. **(E)** Volcano plot of differential gene expression between HAS and LAS neutrophils. **(F)** Upset analysis of the intersection of highly expressed genes in HAS neutrophils and the four modules associated with HAS.

To investigate the gene modules associated with neutrophil subgroups, hdWGCNA was used for weighted gene co-expression network analysis. The power value was set to 7 based on the scale-free topology criterion (**Figure 5A**). This analysis identified 10 distinct gene modules, which were subsequently clustered using hierarchical clustering, and the resulting dendrogram was visualized (**Figures 5B, C**). The expression levels of these gene modules were assessed in the three neutrophil subgroups. Notably, DTAS neutrophils exhibited high module scores across all identified gene modules, indicating a more uniformly active gene expression profile (**Figure 5D**). In contrast, when comparing HAS and LAS neutrophils, specific gene modules showed differential expression. Pink, brown, green, and mengtA modules were expressed at significantly higher levels in HAS neutrophils compared to LAS neutrophils (**Figure 5D**). Differential gene expression analysis between HAS and LAS neutrophils revealed several key genes that were more highly expressed in HAS neutrophils (**Figure 5E**). To further investigate the functional implications of these findings, Upset analysis was performed to identify the intersection of HAS-specific genes and the top four gene modules associated with HAS neutrophils. This analysis revealed a set of core genes that are highly expressed in HAS neutrophils and are linked to the four identified gene modules (**Figure 5F**).

### Predictive gene identification for neutrophil polarization linked to arginine metabolism

**Figure 6:**
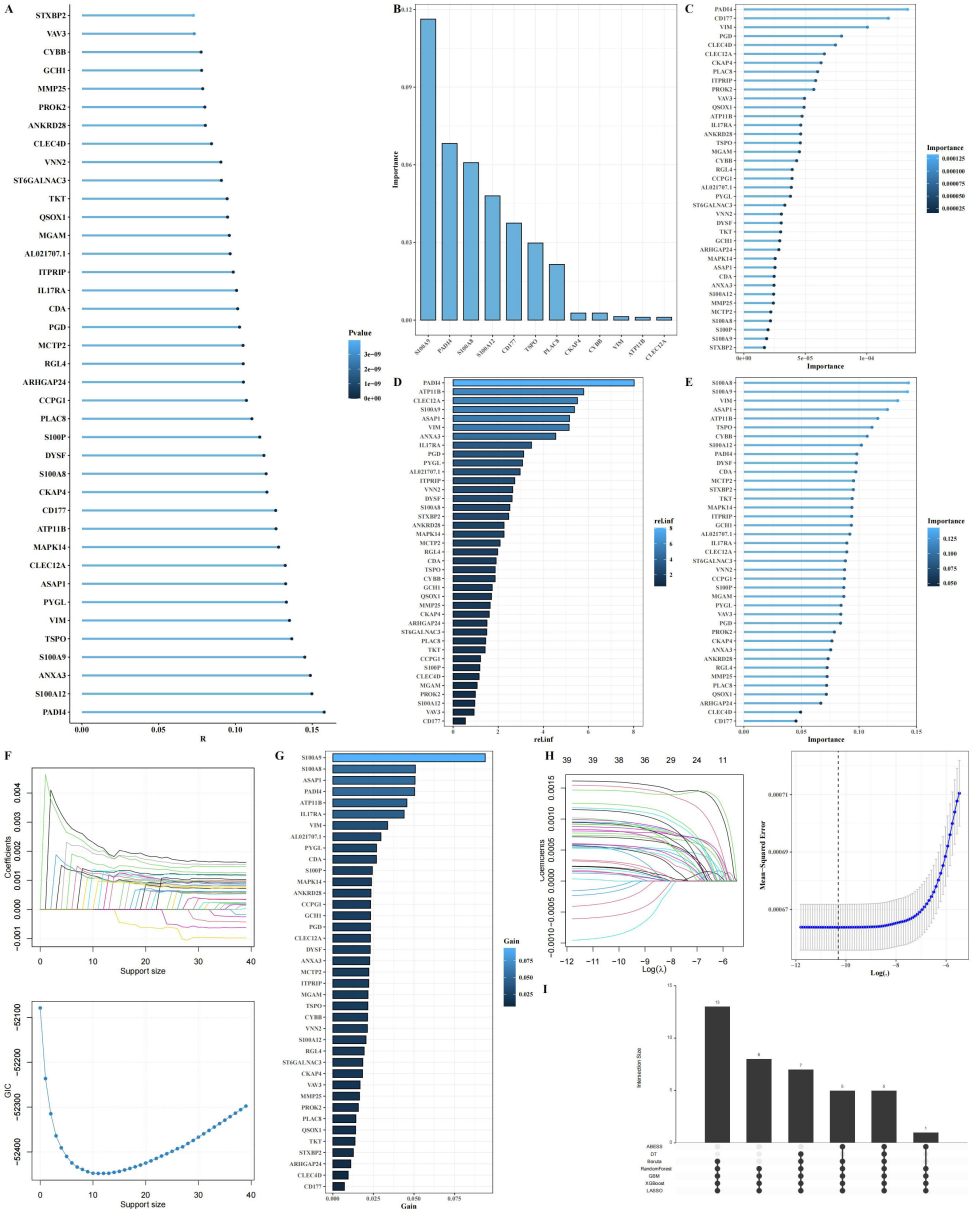
Identification of key arginine metabolism-related genes regulating neutrophil function through machine learning. **(A)** Lollipop plot showing the correlation between 39 intersecting genes and arginine metabolism scores. **(B-H)** Importance ranking of genes using seven machine learning algorithms (Decision Trees, Random Forest, GBM, Boruta, ABESS, XGBoost, and LASSO). **(I)** Upset plot of the intersecting genes selected across the seven machine learning algorithms.

From the 64 genes identified through the intersection of hdWGCNA modules and differential gene expression analysis, we further filtered for genes most significantly associated with arginine metabolism. A total of 39 genes were selected based on their correlation with arginine metabolism scores (**Figure 6A**). To identify key predictive genes, we applied seven machine learning algorithms: Decision Trees, Random Forests, GBM, Boruta, ABESS, XGBoost, and LASSO (**Figures 6B–H**). Each algorithm ranked the importance of genes in relation to arginine metabolism. An Upset analysis was then performed on the gene sets identified by each algorithm, revealing five intersecting genes that were consistently identified across all algorithms (**Figure 6I**).

### ATP11B and PADI4 as potential biomarkers

**Figure 7:**
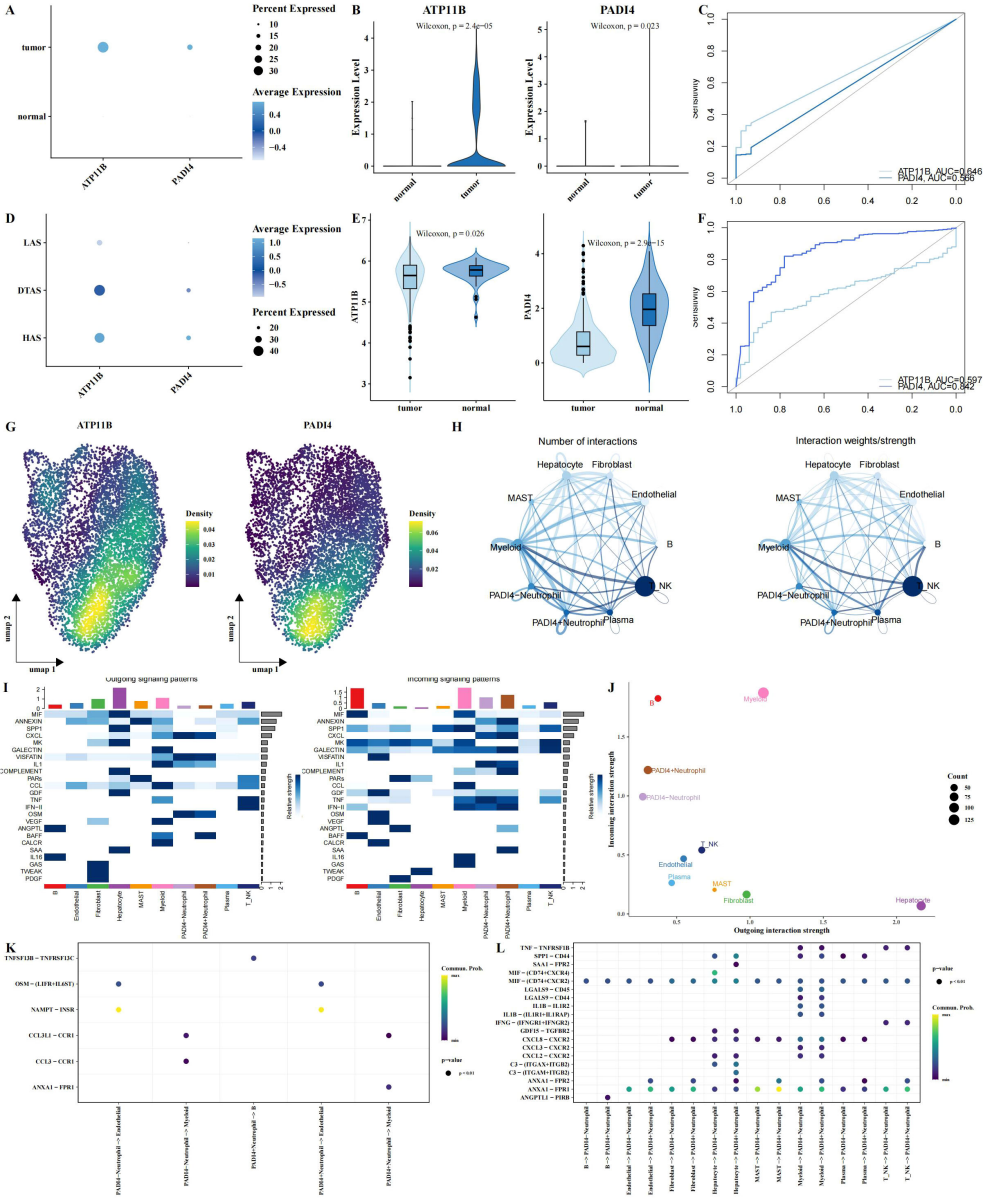
Multi-omics analysis of ATP11B and PADI4 expression and neutrophil polarization. **(A–C)** Expression patterns of ATP11B and PADI4 across different tissue types, visualized using bubble plots **(A)**, violin plots **(B)**, and ROC curve analysis for tissue classification **(C)**, all based on single-cell RNA sequencing data. **(D)** Expression differences of ATP11B and PADI4 between neutrophil subgroups using bubble plots. **(E, F)** Expression levels of ATP11B and PADI4 in the TCGA dataset, shown through violin plots and ROC curve analysis, with tissue type classification results. **(G)** UMAP plot showing the probability density distribution of ATP11B and PADI4 expression specifically in neutrophils from the single-cell RNA sequencing data. **(H)** Communication networks between neutrophils (stratified by PADI4 expression, PADI4+ vs. PADI4-) and other cell types. **(I)** Heatmaps illustrating outgoing (left) and incoming (right) signaling patterns across different cell types, showing the differential signaling activity between PADI4+ and PADI4- neutrophils and their interactions with surrounding cells. **(J)** Scatter plot comparing outgoing interaction strength (x-axis) versus incoming interaction strength (y-axis) for each cell type, illustrating how neutrophil subgroups (PADI4+ and PADI4-) differ in their communication dynamics. **(K, L)** Bubble plots illustrating ligand-receptor communication between PADI4+ and PADI4- neutrophils and other cell types.

Among the five intersecting genes identified, ATP11B and PADI4 have been extensively reported for their roles in tumors. To explore their expression dynamics, we first analyzed the expression of these genes in tumor and adjacent normal tissues using single-cell RNA sequencing data. Our results revealed a significant upregulation of both genes in tumor tissues (**Figures 7A–C**), with the lowest expression observed in the low arginine state (LAS) neutrophil subgroup (**Figure 7D**). However, in the TCGA dataset, the expression of these genes in tumor tissues was significantly decreased (**Figures 7E, F**). This discrepancy suggests that bulk tissue analysis may not fully capture the nuanced expression changes of these genes in distinct cellular contexts. By examining scRNA-seq data, we observed considerable variation in the expression of ATP11B and PADI4 within neutrophil populations, highlighting the importance of single-cell resolution for understanding these changes (**Figure 7G**). Using PADI4 expression as a marker, we separated neutrophils into PADI4+ and PADI4- subgroups for further analysis. We performed cell-cell communication analysis using the CellChat package and identified significant differences in the communication networks between the two subgroups (**Figures 7H, I**). Specifically, PADI4+ neutrophils received more external signaling compared to PADI4- neutrophils (**Figure 7J**). Additionally, communication pathways between PADI4+ neutrophils and other cell types showed distinct patterns. PADI4+ neutrophils transmitted stronger ANXA1 − FPR1 signals to myeloid cells, whereas PADI4- neutrophils were more involved in transmitting CCL3 − CCR1 signals to myeloid cells (**Figure 7K**). Furthermore, significant differences were observed in signal reception between the two subgroups, especially in their interaction with mast cells (**Figure 7L**).

### Functional validation of PADI4 in LIHC cell lines

**Figure 8:**
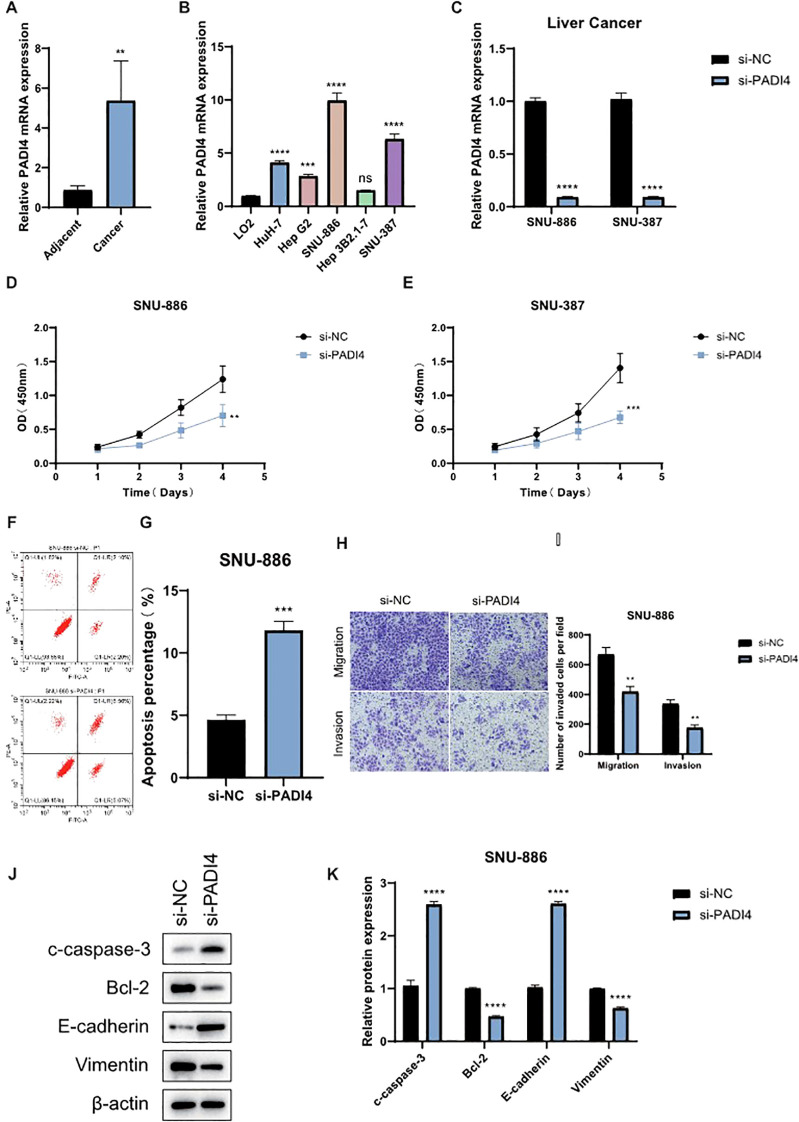
Functional Validation of PADI4 in LIHC. **(A)** qPCR analysis showing the expression levels of PADI4 in LIHC tumor tissues compared to adjacent normal tissues. **(B)** Comparison of PADI4 expression in different LIHC cell lines (HuH-7, Hep G2, SNU-886, Hep 3B2.1-7, SNU-387) relative to the normal hepatocellular cell line LO2. **(C)** Validation of PADI4 knockdown in SNU-886 and SNU-387 LIHC cell lines by qRT-PCR. **(D, E)** CCK-8 proliferation assay demonstrating significantly reduced cell proliferation in PADI4-depleted LIHC cells compared to control cells. **(F, G)** Flow cytometry analysis showing a significant increase in apoptosis in PADI4-knockdown SNU-886 cells. **(H, I)** Transwell migration and invasion assays revealing a significant reduction in both migration and invasion potential in PADI4-silenced LIHC cells compared to controls. **(J, K)** Western blot analysis confirming that PADI4 knockdown results in reduced Bcl-2 expression, increased cleaved caspase-3, and upregulation of E-cadherin, while downregulating Vimentin, further supporting the role of PADI4 in regulating apoptosis and epithelial-mesenchymal transition (EMT) in LIHC. Asterisks denote statistical significance as follows: **P < 0.01; ***P < 0.001; ****P < 0.0001; "ns" indicates not significant (*P* ≥ 0.05).

To further investigate the role of PADI4 in LIHC, we first analyzed its expression in tumor and adjacent normal tissues from clinical LIHC patient samples. qPCR analysis revealed that PADI4 expression was significantly elevated in tumor tissues compared to adjacent normal tissues (**Figure 8A**). Subsequently, we examined PADI4 expression in various liver cell lines and found that SNU 886 and SNU387 cells exhibited notably higher PADI4 levels compared to the normal liver cell line, LO2 (**Figure 8B**). Based on these findings, we selected SNU 886 and SNU387 cells for further functional studies. To assess the functional impact of PADI4, we knocked down its expression in both cell lines using siRNA. qPCR confirmed the successful depletion of PADI4 mRNA in the si-PADI4 groups, with expression significantly reduced compared to the negative control group (si-NC; *P*<0.0001) (**Figure 8C**). This knockdown led to a marked reduction in cell proliferation, as evidenced by CCK-8 assay results, indicating that PADI4 contributes to cell growth in LIHC (**Figures 8D, E**). Flow cytometry analysis further demonstrated the effect of PADI4 knockdown on cell apoptosis. Cells with silenced PADI4 exhibited a significant increase in apoptosis rate compared to the control group (*P*<0.0001), suggesting that PADI4 may play a role in inhibiting apoptotic processes in LIHC (**Figures 8F, G**). Additionally, Transwell migration and invasion assays revealed that PADI4 knockdown significantly reduced both cell migration and invasion capacities. The number of cells migrating to the lower chamber of the Transwell insert was considerably lower in the si-PADI4 group compared to the si-NC group (*P*<0.0001), and similarly, fewer cells invaded through the membrane (*P*<0.01) (**Figures 8H, I**). Western blot analysis confirmed the molecular effects of PADI4 knockdown at the protein level. As expected, PADI4 protein expression was significantly reduced in the si-PADI4 group (*P*<0.05). In addition, we observed changes in apoptosis and epithelial-mesenchymal transition (EMT) markers: the pro-apoptotic protein c-caspase-3 was upregulated, while the anti-apoptotic protein Bcl-2 was downregulated (*P*<0.05). Furthermore, PADI4 knockdown resulted in an upregulation of E-cadherin and a downregulation of Vimentin (*P*<0.05), indicating that PADI4 depletion may inhibit the EMT process, further supporting its role in tumor progression and metastasis (**Figures 8J, K**).

## Discussion

In this study, we identify three distinct neutrophil subgroups—HAS, DTAS, and LAS—based on their arginine metabolism profiles in the LIHC TME. Our analysis reveals that LAS neutrophils, characterized by low arginine metabolism, are predominantly enriched in tumor tissues and display a more undifferentiated, immunosuppressive phenotype. In contrast, HAS neutrophils, with higher arginine metabolism, are more differentiated and primarily located in normal tissues, suggesting a potentially anti-tumor role. Additionally, we identify ATP11B and PADI4 as key genes involved in regulating neutrophil polarization, providing new insights into the metabolic reprogramming that drives neutrophil functional divergence in LIHC.

Arginine metabolism in LIHC presents a paradox: while tumor cells actively accumulate arginine, the TME is functionally arginine-deprived (37). Our scRNA-seq data reveal significantly lower arginine metabolism scores in tumor tissues—particularly in hepatocytes, fibroblasts, and myeloid cells—consistent with the repression of the urea cycle and the development of arginine auxotrophy in tumors (38, 39). However, tumor cells themselves utilize compensatory mechanisms, such as RBM39-mediated asparagine synthesis, to maintain high intracellular arginine levels (40). This metabolic imbalance likely reflects the spatial heterogeneity within the TME, where tumor cells sequester arginine through the transporter SLC7A1, while stromal and immune cells, particularly neutrophils in the LAS subgroup, face arginine depletion (41). This depletion drives immune suppression and contributes to a pro-tumor environment.

Our study further underscores the crucial role of arginine metabolism in neutrophil polarization within the LIHC TME. We observed that neutrophils can be classified into three subgroups based on their arginine metabolism profiles: HAS, DTAS, and LAS. LAS neutrophils, characterized by low arginine metabolism, are predominantly found in tumor tissues and exhibit an undifferentiated phenotype, with reduced differentiation potential as indicated by lower CytoTRACE scores. This aligns with observations that neutrophils in the TME, similar to myeloid-derived suppressor cells (MDSCs), deplete extracellular arginine to suppress T-cell function (42). These LAS neutrophils likely contribute to immune suppression and tumor progression by creating an environment conducive to immune evasion.

In contrast, HAS neutrophils, with higher arginine metabolism, are more mature and enriched in normal tissues. These neutrophils likely retain anti-tumor potential, as they show increased expression of markers associated with pro-inflammatory responses, such as nitric oxide (NO) production. NO is critical for their anti-tumor activity, as it enhances phagocytosis and promotes NET formation (43). Thus, a balance exists between the iNOS-driven pro-inflammatory responses of HAS neutrophils and the ARG2-dependent immune suppression in LAS neutrophils. Our data suggest that arginine metabolism serves as a key determinant in neutrophil functional polarization, where extracellular arginine availability influences whether neutrophils adopt a pro-tumor or anti-tumor phenotype.

ATP11B and PADI4 emerge as key regulators of neutrophil polarization in the LIHC TME. While the role of ATP11B in neutrophil polarization has been less explored, there is evidence suggesting that ATP11B may enhance T-cell function by upregulating and externalizing S1PR1 (44), a mechanism that could similarly affect neutrophil function. PAD4, on the other hand, plays a critical role in the formation of NETs (45), which have been implicated in promoting liver cancer progression and metastasis. Together, ATP11B and PADI4 not only serve as potential biomarkers for neutrophil functional modulation but also highlight the complex interplay between metabolic reprogramming and immune polarization in the TME. Their differential expression in neutrophil subgroups provides new insights into how metabolic pathways shape immune cell behavior, opening up novel therapeutic strategies aimed at reprogramming the immune microenvironment for more effective cancer treatments.

### Limitations and future directions

Despite the valuable insights from this study, several limitations should be addressed in future research. First, our analysis relied on publicly available datasets, which may not fully reflect the complexity of individual patient tumor microenvironments. Validation using clinical samples or patient-derived models is needed. Second, while we focused on arginine metabolism, other metabolic pathways and immune cell interactions in the tumor microenvironment remain unexplored. Expanding these investigations will provide a more comprehensive understanding of tumor progression. Although we observed that arginine metabolism influences neutrophil phenotypes, our study does not fully clarify how it affects neutrophil function, warranting further research. Additionally, scRNA-seq has limitations in detecting low-abundance transcripts, which may affect the sensitivity of identifying key regulatory molecules. Future studies incorporating advanced technologies or complementary methods may help address this limitation. Finally, longitudinal studies and clinical trials are crucial to evaluate the potential of biomarkers like PADI4 in predicting disease progression and therapeutic response in LIHC.

## Conclusion

In conclusion, our study highlights the critical role of arginine metabolism in neutrophil polarization within the LIHC TME. By influencing neutrophil differentiation and immune function, metabolic reprogramming serves as a key regulatory mechanism in tumor progression and immune evasion. Our findings suggest that targeting arginine metabolism could offer a promising therapeutic strategy to modulate neutrophil function and improve cancer treatment outcomes. Further exploration of metabolic pathways in neutrophils will be essential to optimize strategies aimed at overcoming the immunosuppressive TME and enhancing the efficacy of cancer therapies.

## Data Availability

The original contributions presented in the study are included in the article/**Supplementary Material**. Further inquiries can be directed to the corresponding authors.
