# Supplementary figures and images for: Dysregulated arginine metabolism is associated with pro-tumor neutrophil polarization in liver cancer
Source: Front Immunol. 2025 Oct 23;16:1673665. doi: 10.3389/fimmu.2025.1673665 (PMC12589071; doi:10.3389/fimmu.2025.1673665)

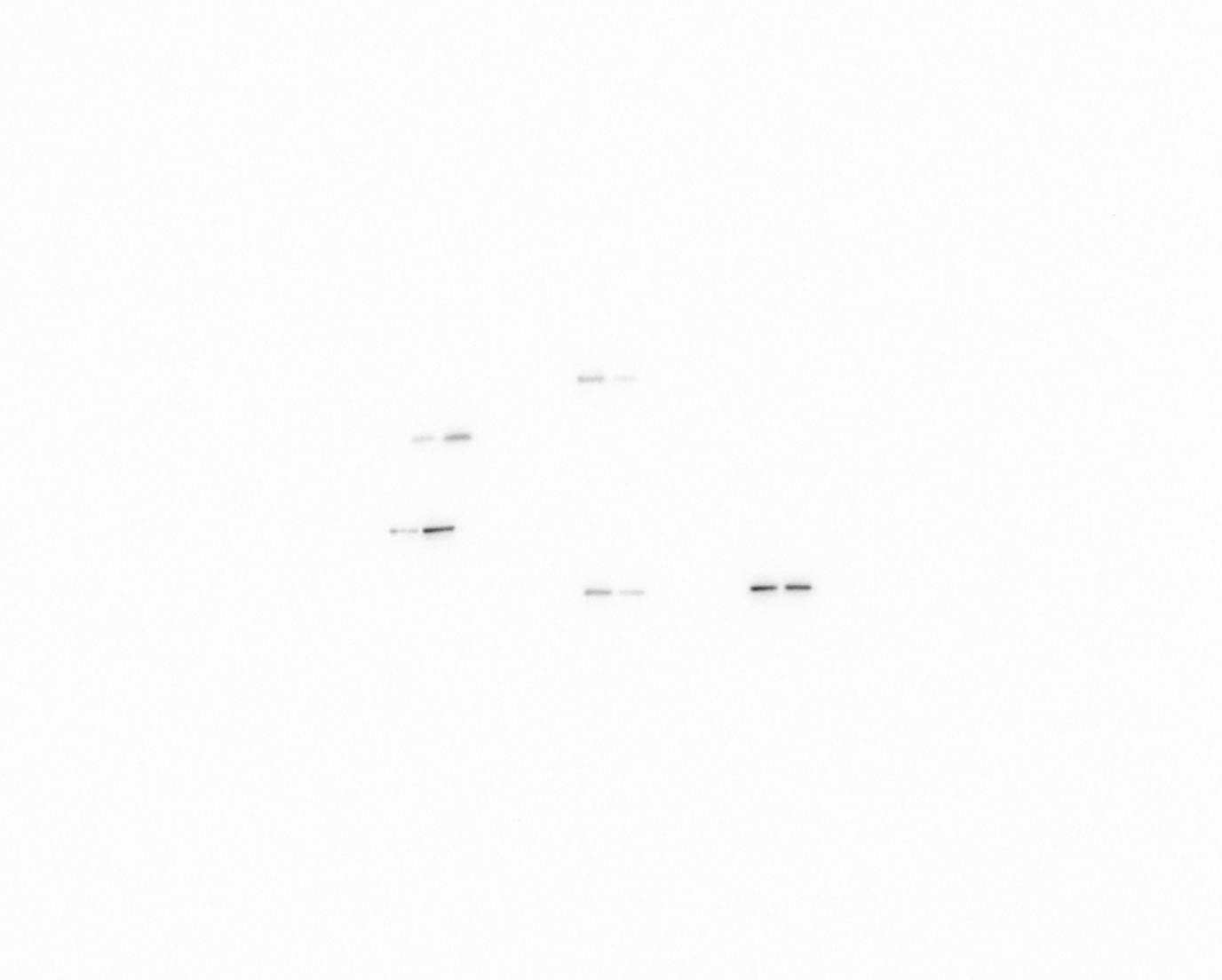

Supplement: Supplementary file 1 [file Image1.tif]

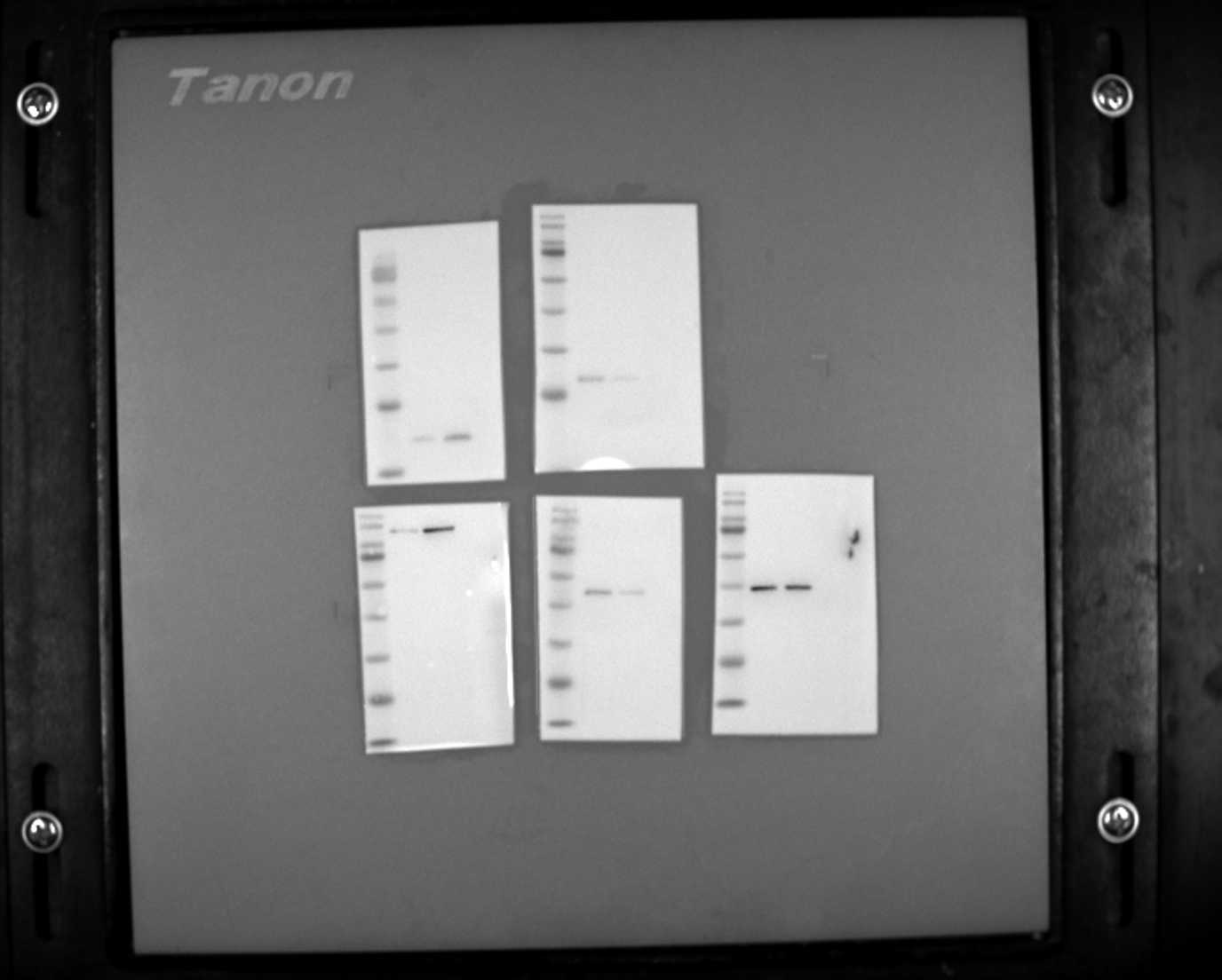

Supplement: Supplementary file 2 [file Image2.tif]

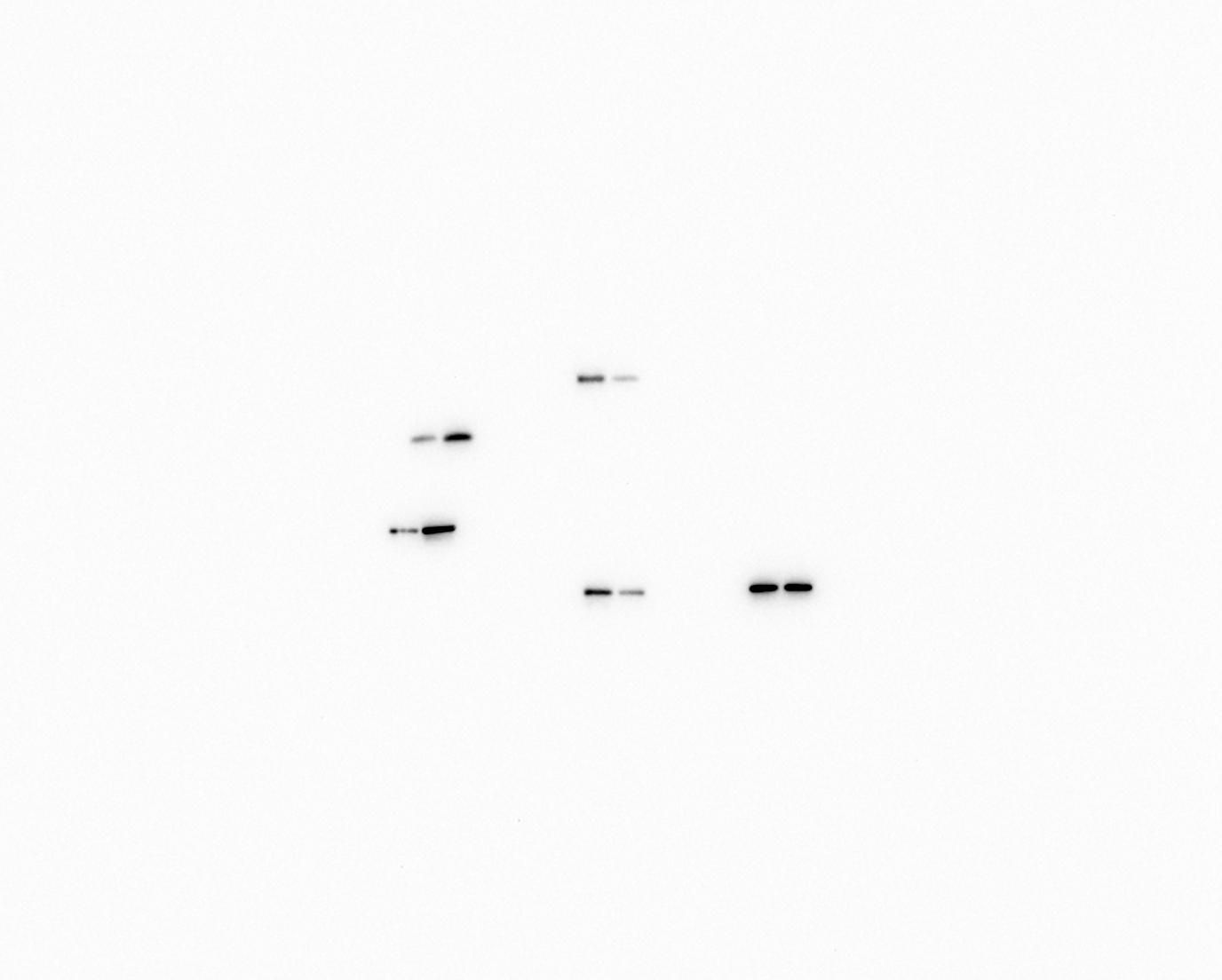

Supplement: Supplementary file 3 [file Image3.tif]

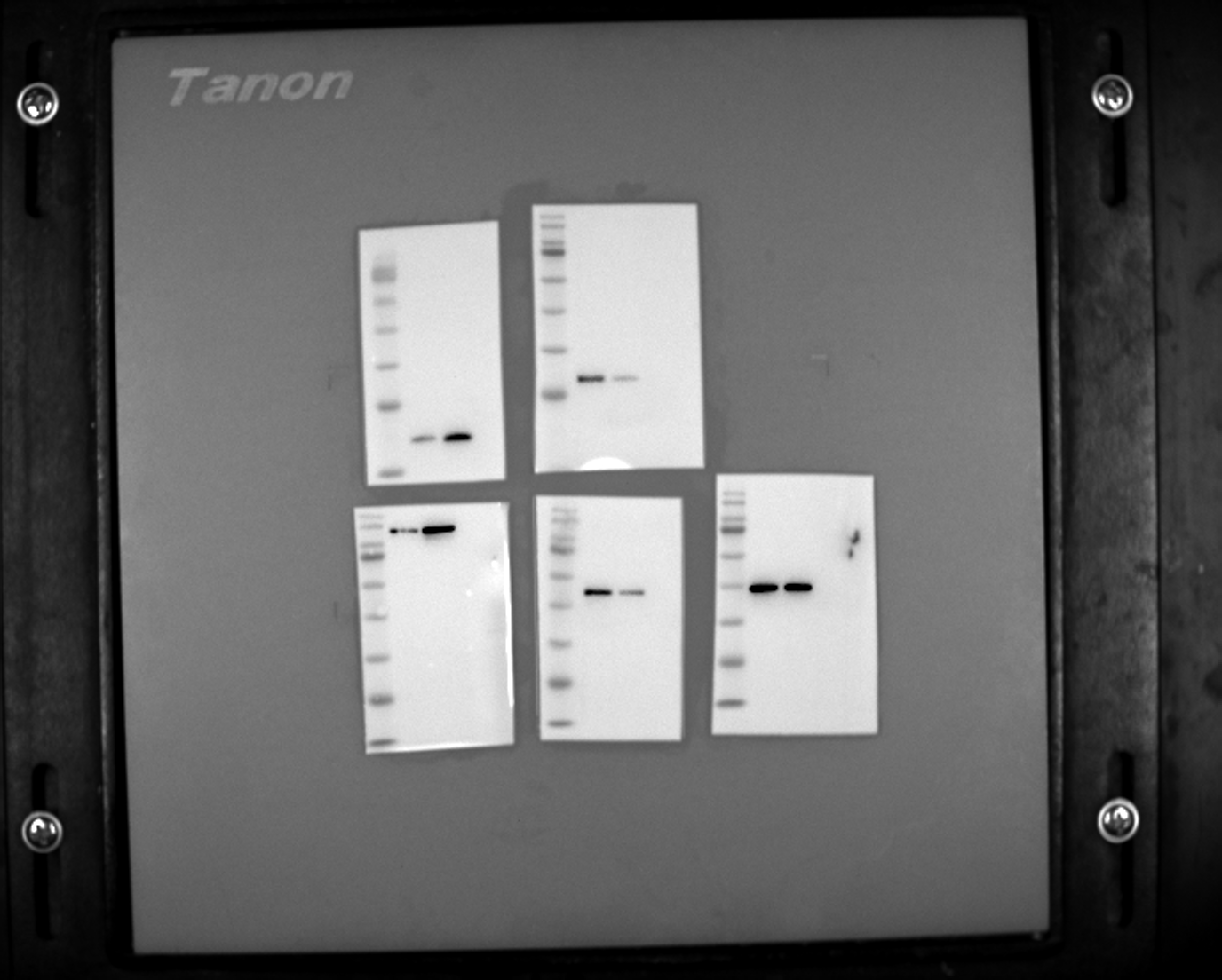

Supplement: Supplementary file 4 [file Image4.tif]

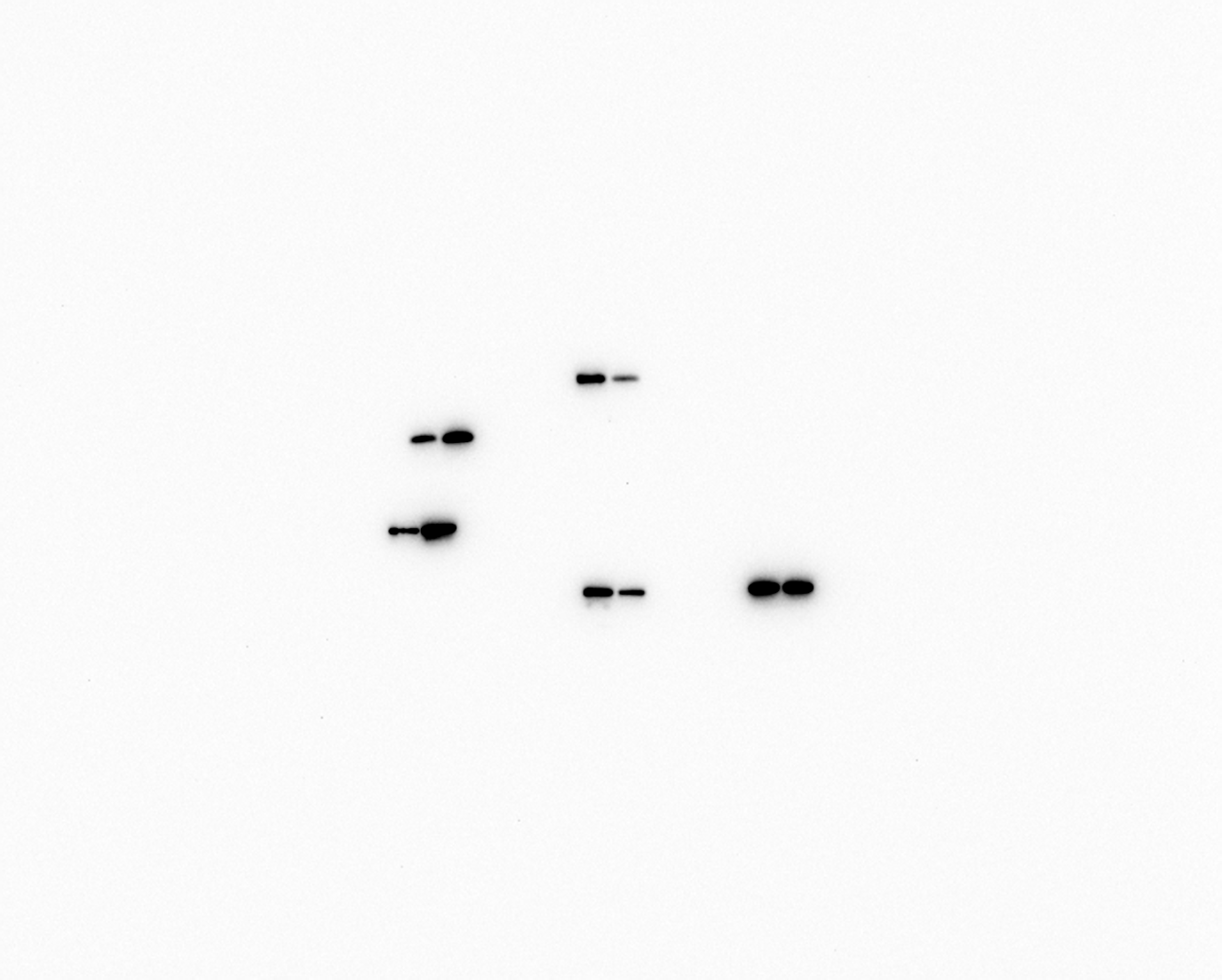

Supplement: Supplementary file 5 [file Image5.tif]

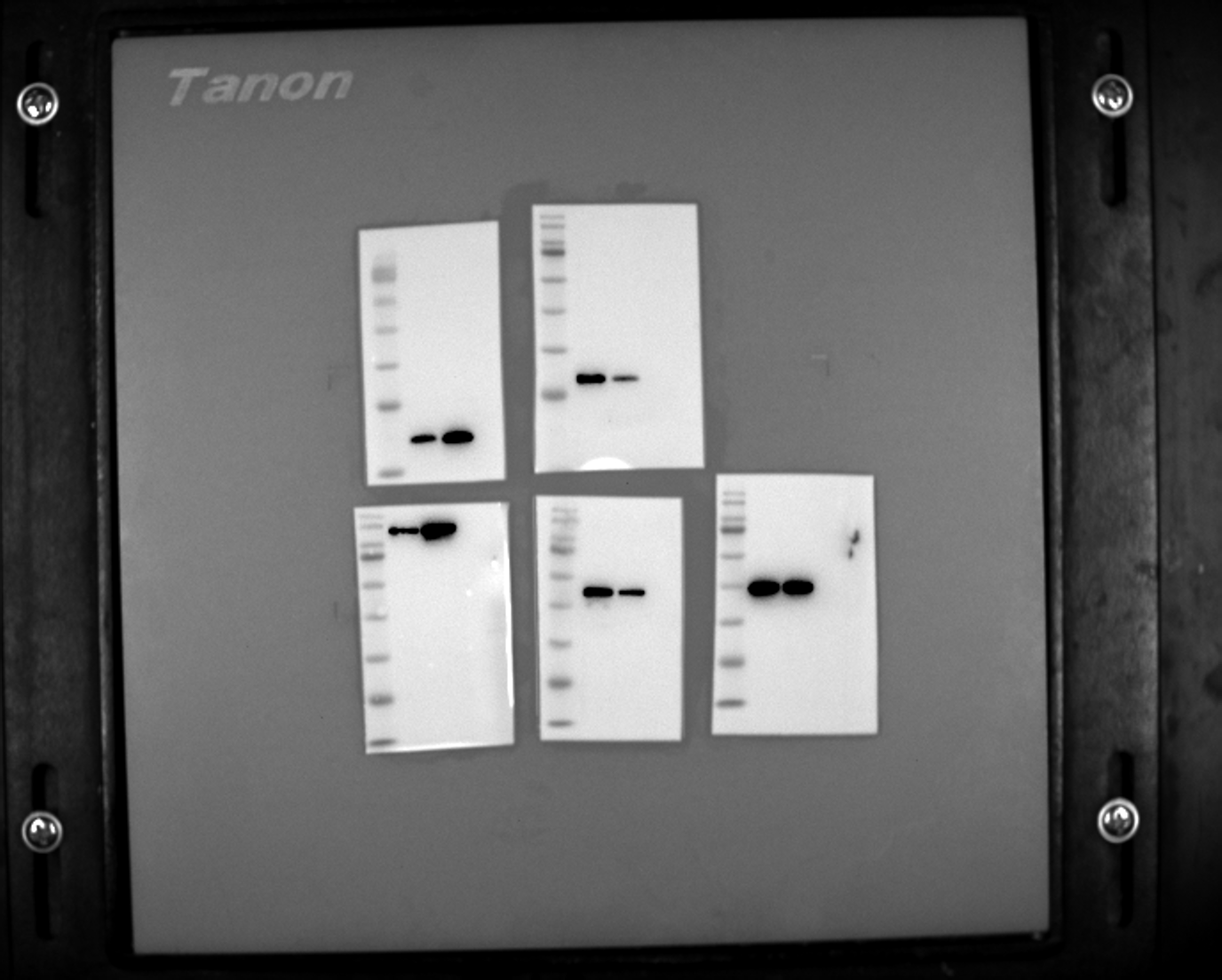

Supplement: Supplementary file 6 [file Image6.tif]
